# Diagnosis of rare diseases based on facial phenotype: a quantitative assessment using 2D and 3D photography in Stickler syndrome

**DOI:** 10.1186/s13023-026-04323-4

**Published:** 2026-07-27

**Authors:** Adèle Rohée-Traoré, Maxime Taverne, Quentin Hennocq, Thomas Bongibault, Alejandra Daruich, Dominique Bremond-Gignac, Jean-Daniel Kün-Darbois, Pierre-Raphaël Rothschild, Roman-Hossein Khonsari

**Affiliations:** 1 Laboratoire Forme et Croissance du Crâne, Institut Imagine, Paris, France; 2 Service de chirurgie maxillo-faciale, Centre Hospitalier Universitaire d’Angers, Angers, France; 3 Service de sciences des données, Institut Imagine, Paris, France; 4 Service d’ophtalmologie, Hôpital Necker - Enfants malades, Assistance Publique - Hôpitaux de Paris, Paris, France; 5 Service d’ophtalmologie - OphtalmoPôle de Paris, Hôpital Cochin - Port Royal, Assistance Publique - Hôpitaux de Paris, Paris, France; 6 Service de chirurgie maxillo-faciale et chirurgie plastique, Hôpital Necker - Enfants malades, Assistance Publique - Hôpitaux de Paris, Paris, France; 7 CRMR MAFACE, Filière TêteCou, ERN CRANIO, Paris, France; 8 Faculté de médecine, Université Paris Cité, Paris, France

**Keywords:** Artificial intelligence, Photogrammetry, Pierre Robin syndrome, Retinal detachment, Stickler syndrome, Supervised machine learning

## Abstract

Stickler syndrome (SS) is a rare genetic disorder caused by mutations in collagen-encoding genes. Patients with SS are at an increased risk of retinal detachment, with ophthalmologic complications often presenting as the first symptom due to significant diagnostic delays. These delays contribute to severe visual impairment, even in early childhood, making diagnostic uncertainty a major clinical challenge. This study assessed the feasibility of early SS detection using facial features. Supervised machine learning models, including XGBoost and support vector machines (SVM), were applied to 2D and 3D facial photographs to differentiate individuals with SS from healthy controls. The models achieved 92% accuracy in identifying SS patients using full-face 2D and 3D photographs. When focusing specifically on the orbitonasal region in 3D images, classification accuracy increased to 96%. Facial morphology in rare diseases such as Stickler syndrome can serve as a highly accurate, non-invasive screening tool. AI-based analysis has the potential to reduce diagnostic delays and prevent associated complications, improving patient outcomes.

## Introduction

Stickler syndrome (SS) is a rare genetic disorder affecting approximately 1 in 7,500 to 9,000 births, caused by mutations in genes encoding collagens [1]. The most frequently reported variants involve collagen types II, IX, and XI, including *COL2A1*, *COL9A1*, *COL9A2*, *COL9A3*, *COL11A1*, and *COL11A2*. Additionally, mutations in *BMP4* and *LOXL3* have been reported [1, 2]. SS is typically inherited in an autosomal dominant manner, although autosomal recessive inheritance is also possible [1]. Although genotype–phenotype correlations remain limited in Stickler syndrome, with marked intra- and inter-familial variability, the condition is classically subdivided into subtypes based on the affected gene, each of which is associated with a preferential phenotypic expression [2]. SS type 1 is caused by *COL2A1* variants, it is the most prevalent form, accounting for approximately 80–90% of cases. Its manifestations are predominantly ophthalmological and include strabismus, vitreous architectural abnormalities, congenital myopia, glaucoma, cataracts, and a significantly increased risk of retinal detachment [1, 3]. Type 1 SS may present as a purely ocular form or be associated with additional systemic features, including middle- and inner-ear anomalies leading to conductive and/or sensorineural hearing loss [4], as well as skeletal abnormalities such as ligamentous hyperlaxity predisposing to early-onset osteoarthritis and scoliosis [1]. Patients may present with a cleft palate, which may be isolated or occur as part of Pierre Robin sequence in approximately 25% of cases [2]. In cases of delayed diagnosis, ophthalmological complications—particularly retinal detachment—are often the initial presenting features. Although retinal detachment most commonly occurs between the ages of 10 and 30, it is also frequently observed before the age of 10 and may lead to severe visual impairment in young children [2, 5, 6]. SS type 2, associated with *COL11A1* variants, combines ocular manifestations—including membranous congenital vitreous anomaly, retinal detachment, and congenital megalophthalmos—with extra-ocular features such as hearing loss, arthropathy, and possible cleft palate [2]. SS type 3, related to *COL11A2* variants, is a systemic form characterized by the absence of ocular involvement. SS types 4, 5 and 6, caused by variants in *COL9A1*, *COL9A2*, and *COL9A3*, respectively, are associated with an increased risk of sensorineural hearing loss, spondyloepiphyseal dysplasia, myopia, vitreoretinopathy, and retinal detachment [1, 2]. Beyond ocular manifestations, Stickler syndrome lacks specific clinical markers, and diagnosis is often established only after the occurrence of one or more visual complications. To date, no universally accepted morphological diagnostic criteria exist, making genetic confirmation essential for definitive diagnosis [1]. Nevertheless, Stickler syndrome subtypes are frequently associated with maxillofacial anomalies. In addition to cleft palate, affected individuals may present with nasal and midface hypoplasia, an elongated philtrum, and micrognathia and/or mandibular retrognathia [7]. The combination of these features, present to varying degrees, may confer a characteristic facial appearance [1, 7]. For experienced clinicians, the so-called ‘Stickler face’ can be highly suggestive when accompanied by other relevant clinical signs. However, the absence of standardized morphological diagnostic criteria makes early recognition challenging, increasing the reliance on genetic testing [1]. This diagnostic uncertainty may delay proper management and lead to severe, irreversible ophthalmologic complications. Therefore, objective measures of the typical ‘Stickler face’ are needed to detect early phenotypic deviations from normal facial development.

This study aims to: (I) identify the key discriminative facial morphological features of SS; (II) describe facial shape variations in SS and healthy controls over time; and (III) lay the foundation for automated screening tools to facilitate early SS detection.

We applied geometric morphometrics to 2D clinical photographs and 3D facial surfaces obtained through photogrammetry, using machine learning classification algorithms to assess the diagnostic specificity and sensitivity of various clinical features. Additionally, we analyzed the natural history of the facial phenotype in SS to evaluate the relevance of these screening criteria concerning age at presentation.

## Materials and methods

### Ethical clearance

This study was approved by the CESREES (Comité Éthique et Scientifique pour les Recherches, les Études et les Évaluations dans le domaine de la Santé, №4570023bis) and the CNIL (Commission Nationale Informatique et Libertés, №MLD/MFI/AR221900). It was conducted in accordance with the 1964 Declaration of Helsinki and its subsequent amendments. Informed written consent was obtained from the legal representatives of each child or from the patients themselves if they were of legal age.

### Inclusion criteria and data acquisition

#### 2D photographs

Photographs were obtained from a large database maintained by the Department of Maxillofacial and Plastic Surgery at Necker-Enfants Malades Hospital. This database contains over 1,000,000 images of more than 30,000 patients, with records dating back to 1979. All photographs taken since 1995 were captured by a professional medical photographer using a Nikon D7000 camera under standardized conditions. Images from 1979 to 1995, originally non-digitized, were scanned using an Epson Perfection V850 Pro scanner.

Photographs from 1979 to 2019 were included retrospectively, while images from 2019 to 2023 were included prospectively for patients with genetically confirmed Stickler syndrome. Clinical data were retrieved from the local data warehouse Dr Warehouse [8]. The validation set was constructed using the same inclusion and exclusion criteria as the training set, ensuring no overlap between patients in the training and validation groups. Data on age at the time of the photograph and gender were also collected.

#### 3D photogrammetry

All patients under 18 years of age with genetically confirmed Stickler syndrome who were clinically assessed in the Ophthalmology or Maxillofacial and Plastic Surgery departments at Necker-Enfants Malades Hospital (Assistance Publique - Hôpitaux de Paris) between January and July 2023 were included in the study.

Age- and sex-matched healthy controls were selected, with their facial surfaces extracted from CT scan data. Control CT scans were performed for benign conditions such as trauma or infections, ensuring no craniofacial anomalies. Patients with a history of facial surgery *(*rhinoseptoplasty, blepharoplasty, orthognathic surgery, genioplasty*)* or maxillofacial fractures were excluded.

Facial surfaces were extracted following soft-tissue segmentation using 3DSlicer v.5.6.0 [9]. For SS patients, 3D surface scans were obtained using an Artec EVA handheld scanner (*Artec 3D*,* Luxembourg*) and processed into a 3D mesh using Artec Studio 13 Professional (*Artec 3D*,* Luxembourg*,* 2018*).

### Data processing

#### 2D photographs

Landmarking, Procrustes superimposition, Principal Component Analysis (PCA), and textural analysis were performed on 14 key facial regions using the Gray-Level Co-occurrence Matrix (GLCM) method, as previously described [10]. Geometric features (Procrustes coordinates) and textural features (GLCM) were combined for further analysis.

#### 3D photogrammetry

Due to the specific morphology of the facial region leading to the absence of repeatable anatomical landmarks on smooth surfaces (e.g., cheeks), a template-based registration method was used to align all 3D facial surfaces. This process was conducted using Faceform Wrap (*Faceform v. 2023.11.4*,* Yerevan*).

A standard symmetric facial mesh consisting of 2,242 elements (from the Faceform library) was used as a template. Alignment was performed through: (I) Rigid Iterative Closest Point (ICP) registration, followed by (II) Non-rigid Iterative Closest Point (NICP) registration, which minimized bending energy while preserving the original facial shape.

**Fig. 1:**
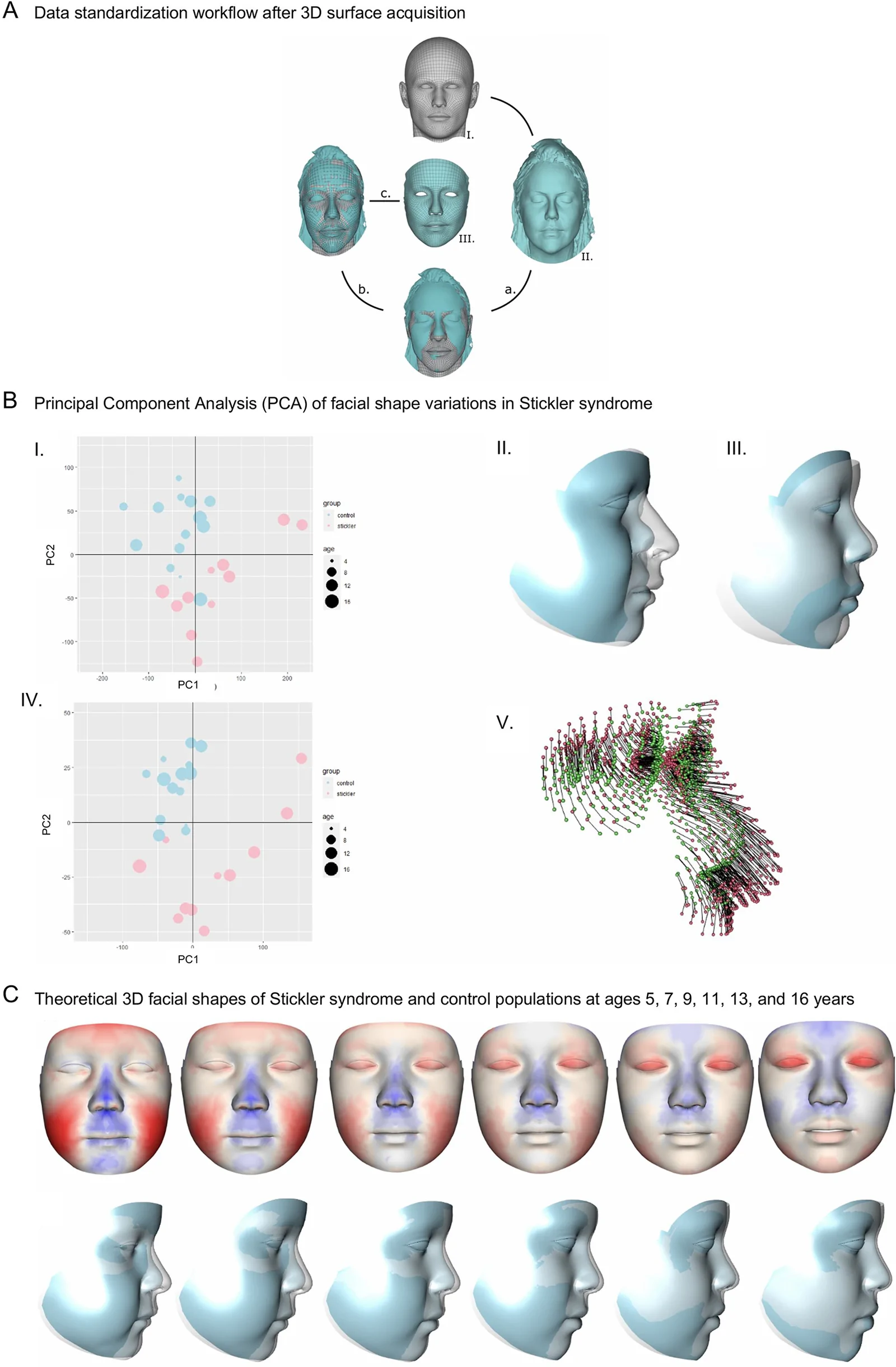
3D facial data standardization and analysis. (**A**) Based on several facial landmarks, the standardized face template (I) and the raw face scan of each participant (II) were first rigidly aligned using rigid Iterative Closest Point (ICP) procedure (a.). Non-rigid Iterative Closest Point (NICP) registration was then applied (b.). Finally, only the nodes corresponding to the facial region of interest were kept (c.), resulting in a final wrapped face (III) presenting the exact same shape as II., while preserving the node topology of I. This process was repeated for each participant, ultimately providing a new set of wrapped shapes which nodes 3D coordinates were extracted and used in the subsequent analyses. (**B**) Principal Component Analysis (PCA) of facial shape variations in Stickler syndrome. (I) PCA plot of the full face. (II-III) 3D reconstructions of extreme values along PC1(II) and PC2 (III). (IV) PCA plot of the orbitonasal region. (V) 3D reconstructions associated with extreme PC1 values (Pink: Stickler syndrome; Blue: Controls). (**C**) Theoretical 3D facial shapes of Stickler syndrome and control populations at ages 5, 7, 9, 11, 13, and 16 years. Superimposing the theoretical age-specific shapes highlights developmental anomalies associated with Stickler syndrome over time. The faces represent the theoretical shapes of Stickler patients at 5, 7, 9, 11, 13, and 16 years of age (from left to right), with protrusive areas shown in red and retrusive areas shown in blue relative to the control population. The profiles represent the superimposition of the theoretical shapes of Stickler patients (blue) and control patients (transparent) at ages 5, 7, 9, 11, 13, and 16 years

This process transformed each raw 3D surface into a comparable 3D mesh, ensuring uniform element arrangement and topology (Fig. 1A). The 3D coordinates of all wrapped faces were exported and combined into a single matrix.

To ensure final rigid alignment, Procrustes superimposition was applied using the ProcSym function from the Morpho package [11] in R (v. 4.2.3, R Core Team, Vienna). The resulting Procrustes coordinates were used as input shape data for all subsequent analyses.

### Statistical analyses

#### 2D facial shape

To account for metadata (*age*,* gender*) and non-independence of data (*multiple photographs per patient*), a mixed-effects model was used for each feature. Geometric and textural features were treated as response variables, with age, gender, and ethnicity as explanatory variables, given their significant influence on dysmorphology and diagnosis [12, 13]. A random effect was introduced for age and individual patients.

The model inputs were residuals from linear models for each geometric or textural feature. XGBoost (eXtreme Gradient Boosting) was employed as the supervised machine learning classifier [14], using a tree-based booster with: (I) logistic regression for binary classification, (II) Softmax function for multi-class classification, and (III) 5-fold cross-validation to optimize hyperparameters (learning rate = 0.3, gamma = 0, maximum tree depth = 6).

The final model was applied to an independent test set, and accuracy and AUC were plotted. ROC curves were generated using the plotROC package in R [15].

Two sets of models were developed: (I) diagnostic support – distinguishing SS patients from controls (binary classification) and (II) genotype-phenotype correlation – comparing *COL2A1* vs. *COL11A1* patients and controls.

Additionally, the 2D photograph algorithm was applied to a 30-week-old male fetus photograph with a genetically confirmed Stickler syndrome diagnosis (COL11A2).

#### 3D facial shape

Shape analysis was conducted on the full set of points as well as on five predefined facial regions of interest: nasofrontal, orbital, nasal, orbitonasal, and chin areas. Principal Component Analysis (PCA), implemented using the prcomp function from the stats package in R [16], was applied to identify the primary global and local shape variations within the dataset.

To evaluate whether the facial shape of SS patients differed significantly from that of controls, while accounting for age, a non-parametric Multivariate Analysis of Covariance (MANCOVA) was performed using the procD.lm function from the geomorph package in R [17], with 1,000 permutations. The first Principal Components (PCs) from the PCA, which together explained over 85% of the total variance, were used as dependent variables, with the patient group as the explanatory variable and log-transformed age as the covariate.

Given the well-established influence of age on craniofacial allometric changes, growth trajectories were estimated for each group using two-block Partial Least Squares (2b-PLS) regression, with Procrustes coordinates as block 1 and log-transformed age as block 2 (*pls2B function*,* Morpho package*) [11]. To visualize the morphological variation along the PCs or PLS axes, Thin Plate Spline (TPS) deformation was applied (*tps3d function*,* Morpho package*), and the results were displayed using the shade3d function (*Morpho package*) [11].

**Fig. 2:**
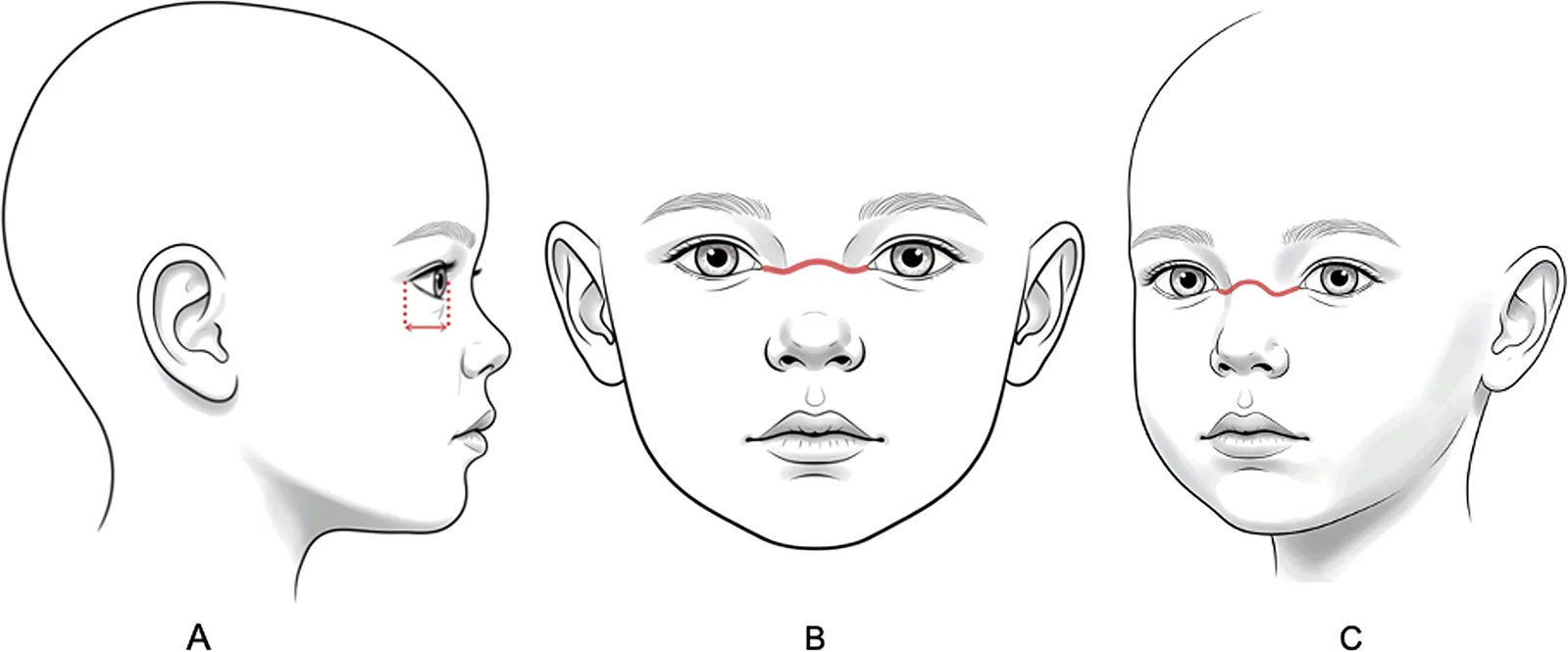
Illustration depicting the linear measurements performed on the 3D surfaces. **A**: ocular protrusion. **B**, **C**: nasal root height

These analyses aimed to identify multivariate morphological features that distinguish SS patients from controls, highlighting deviations in facial growth patterns. To correlate the observed shape variations with clinically accessible, univariate measures, two linear measurements were taken from the orbitonasal region: (I) Ocular protrusion (proptosis) – defined as corneal projection from the external canthus in a strict profile view, measured automatically using the open-access Cliniface software [18]; (II) Nasal root height – defined as the surface distance between the two internal canthi, measured manually using 3DSlicer v.5.6.0 [9] (Fig. 2). This surface distance was calculated as the sum of all node distances spanning the internal canthi, following the convexity of the nasal root. Differences were considered statistically significant at *p* < 0.05.

To classify facial phenotypes, a Support Vector Machine (SVM) classifier was used (*svm function*,* e1071 package*) [19]. Since non-linear classification requires kernel functions to project the data into a higher-dimensional space for optimal linear separation, a kernel-based SVM model was employed. Leave-One-Out Cross-Validation (LOOCV) was used to assess the classification performance of the model. The SVM classifier was first applied to the principal components of the full face and orbitonasal region. It was then further tested using the two linear measurements (nasal root height and proptosis).

## Results

### Cohort

For the 2D photograph training set, a total of 1,166 frontal and lateral facial photographs from 562 patients were included. The mean age was 6.9 ± 3.4 years, ranging from 0 to 22 years, with 52% female participants. The control group consisted of 1,040 photographs from 520 patients, with a mean age of 7.0 ± 4.6 years. The SS group included 126 photographs from 42 patients, with the following genetic distribution: 65% *COL2A1* variants, 32% *COL11A1* variants, and 1% *COL9A1* variants (Table 1 A).

For the validation set, 50 frontal and lateral photographs from 25 patients were included. The control group comprised 38 photographs from 19 patients, while the SS group included 12 photographs from 6 patients. Within the SS group, 3 patients had a *COL2A1* variant, and 3 had a *COL11A1* variant.

For the 3D surface analysis, a total of 25 patients aged 4–16 years (mean age 10.5 ± 3.7 years) were included: 11 SS patients aged 5–16 years (mean age 11 ± 3.2 years) assessed via 3D photogrammetry and 14 control patients aged 4–16 years (mean age 10.1 ± 4.1) with facial surface reconstructions from CT scans (Table 1B). No significant differences were found between SS and control groups in terms of mean age, sex ratio, or ethnicity distribution.

#### 2D shape differences

**Fig. 3:**
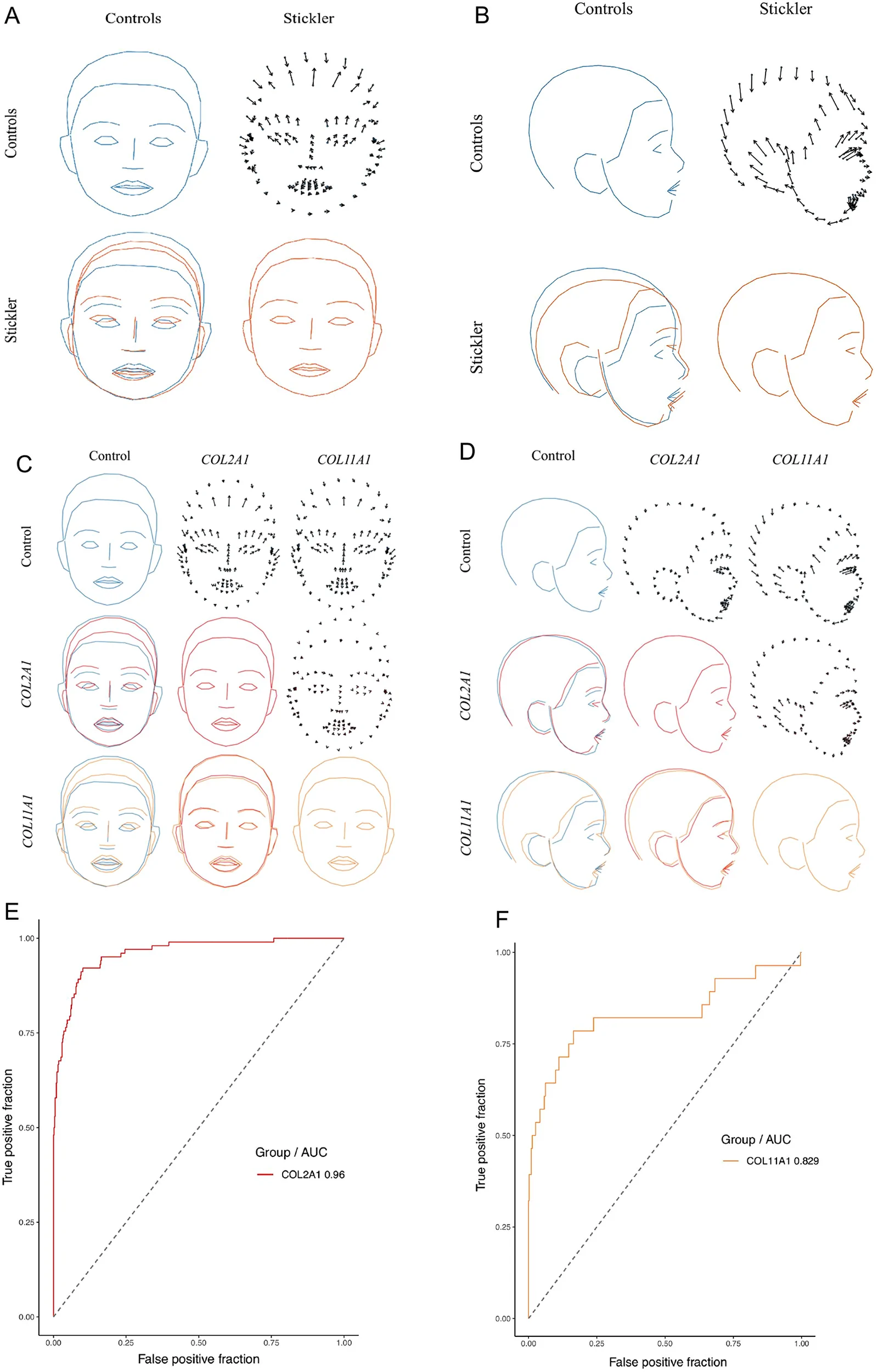
Facial characteristics and genotype-based shape variations in Stickler syndrome. (**A**) Frontal facial features of Stickler syndrome in patients < 5 years old (Blue: Controls; Yellow: Stickler syndrome). (**B**) Lateral facial features of Stickler syndrome in patients < 5 years old (Blue: Controls; Yellow: Stickler syndrome). (**C**) Average facial shapes by genotype groups (frontal view) and Procrustes analysis results for patients < 5 years old (Blue: Controls; Red: COL2A1 variant; Yellow: COL11A1 variant). Diagonal elements (upper left to lower right) represent average facial shapes. Below the diagonal: superimposed average faces from different groups. Above the diagonal: transformation vectors illustrating shape differences between groups. (**D**) Average facial shapes by genotype groups (lateral view) and Procrustes analysis results for patients < 5 years old. Diagonal elements (upper left to lower right) represent average facial shapes. Below the diagonal: superimposed average faces from different groups. Above the diagonal: transformation vectors illustrating shape differences between groups. (**E**) Empirical ROC curves for *COL2A1* vs. controls (training set). (**F**) Empirical ROC curves for *COL11A1* vs. controls (training set)

SS patients exhibited significantly thinner and wider palpebral fissures, hypertelorism, frontonasal hypoplasia (the distance between the palpebral fissure and the frontonasal angle on lateral views, Fig. 3*)* and a longer philtrum. The lower third of the face was characterized by retrognathia (Fig. 3A, B). Ears in SS patients were larger compared to controls but showed no significant differences in shape (Fig. 3B). SS patients were significantly distinguishable from controls in the training set, achieving an accuracy of 0.920 [0.740–0.990] (*p* = 0.041) (Table 2 A). In the validation set, 23 out of 25 patients were correctly classified (Table 2B). Notably, the two misclassified patients carried a *COL11A1* variant. Genotype-specific differences were observed: patients with a *COL2A1* variant exhibited a longer philtrum, whereas retrognathia appeared more severe in the *COL11A1* group (Fig. 3C, D). Patients with a *COL11A1* variant were less distinguishable from controls compared to those with a *COL2A1* variant (Fig. 3E, F, Table 2C). The Area Under the Curve (AUC) was 0.960 for the *COL2A1* group, whereas it was 0.829 for the *COL11A1* group. All patients in the *COL2A1* group were correctly classified, while none in the *COL11A1* group were accurately identified.

**Fig. 4:**
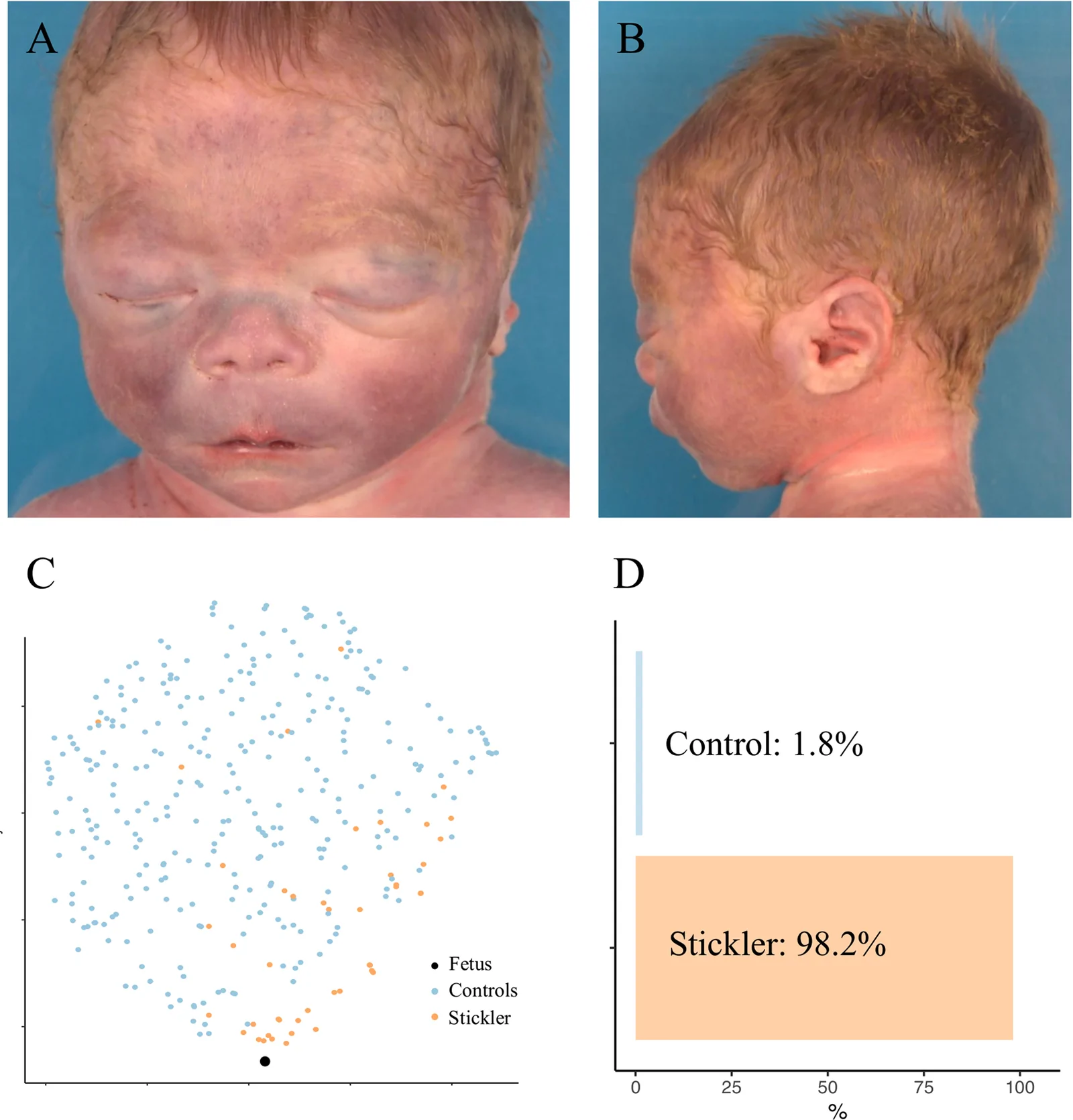
Application of the 2D photograph-based algorithm to a 30-week-old male fetus with a genetically confirmed Stickler syndrome diagnosis (COL11A2). (**A**) Frontal fetal photograph. (**B**) Profile fetal photograph. (**C**) UMAP clustering of the fetus among control and Stickler syndrome patient groups. (**D**) Prediction histogram, indicating a 98.2% probability of Stickler syndrome

For the 30-week-old male fetus photograph (gestational age), the algorithm predicted Stickler syndrome with a probability of 98.2% (Fig. 4).

#### 3D shapes differences

Principal Component Analysis (PCA) of full facial morphology suggested a difference between SS patients and controls (Fig. 1B *– I*). SS patients were characterized by nasal retrusion (*PC1*, Fig. 1B *– II*), mandibular retrognathia (*PC2*, Fig. 1B *– III*), and proptosis (*PC1 and PC2*, Fig. 1B *– II*,* III*). MANCOVA results were significant for both the explanatory variable (group) and the covariate (age), indicating that age was a confounding factor (Table 3).

**Table 1A:** Baseline characteristics of patients and controls in the training set for 2D photographs

		Stickler	Controls	Total
*N*				
	Consultations	63 (11%)	520 (89%)	583
	Photos	126 (11%)	1040 (89%)	1166
	Patients	42 (7%)	520 (93%)	562
Gender				
	Female	23 (37%)	279 (54%)	302 (52%)
	Male	40 (63%)	241 (46%)	281 (48%)
Age (years)				
	Mean ± SD	4.5 **±** 0.7	7.0 **±** 4.6	6.9 **±** 3.4
	Median	2.8	7.1	6.7
	Minimum	0.0	0.2	0.0
	Maximum	14.1	22.1	22.1
Ethnicity				
	Subsaharian african	1 (2%)	28 (5%)	29 (5%)
	Asian	0 (0%)	9 (2%)	9 (2%)
	Caucasian	62 (98%)	483 (93%)	545 (93%)
Genetic variants				
	COL2A1	41 (65%)		41 (65%)
	COL9A1	1 (1%)		1 (1%)
	COL11A1	20 (32%)		20 (32%)

**Table 1B:** Baseline characteristics of stickler syndrome patients and controls for 3D photographs

	Stickler	Controls	Total	*p* value
Patients (*n*)	11	14	25	
Age *(years)*				*> 0.05*
Mean ± SD	11 ± 3.2	10.1 ± 4.1	10.5 ± 3.7	
Min – Max	5–16	4–16	4–16	
Gender				*> 0.05*
Female	3 (27.3%)	7 (50%)	10 (40%)	
Male	8 (72.7%)	7 (50%)	15 (60%)	
Ethnicity				*> 0.05*
Caucasian	7 (63.6%)	8 (57.1%)	15 (60%)	
Subsaharian african	1 (9.1%)	3 (21.45%)	4 (16%)	
Northern african	3 (27.3%)	3 (21.45%)	6 (24%)	

**Table 2A:** Classification performance of 2D-based machine learning models in a binary design (Stickler syndrome vs. controls)

	Value	*p*-value
Accuracy	0.920 [0.740–0.990]	0.041
Sensitivity	1.000	
Specificity	0.667	
Precision	0.905	
Recall	1.000	
F1	0.950	
AUC	1.000	

AUC = Area Under the Curve. F1 = F1-score, harmonic mean of precision and recall

**Table 2B:** Confusion matrix for the binary classification model (Stickler syndrome vs. controls)

		Reference	
		Stickler	Control
Prediction	Stickler	**4**	0
	Control	2	**19**

Bold values indicate true positives

**Table 2C:** Confusion matrix of the model between controls and different subtypes of stickler syndrome (*COL2A1* vs. *COL11A1* vs. controls)

		Reference		
		Control	*COL2A1*	*COL11A1*
Prediction	Control	**19**	0	2
	*COL2A1*	0	**3**	1
	*COL11A1*	0	0	**0**

Bold values indicate true positives

**Table 3:** Results of MANCOVA, using the first principal components (PCs) from PCA, which together explain over 85% of total variance, as dependent variables. The group (Stickler syndrome or control) was used as the explanatory variable, with log-transformed age as the covariate

Variables	Pillai	F	*p*
**A.**			
Group	0.81	9.14	< 0.01
Age	0.68	4.59	< 0.01
Group*Age	0.42	1.58	0.22
**B.**			
Group	0.93	20.61	< 0.01
Age	0.26	0.52	0. 84
Group*Age	0.62	2.32	0.08

The F statistic represents the ratio of variance explained by a given effect to residual variance, quantifying the contribution of a factor (e.g., group, age, or their interaction) to the variability of the dependent variables. Higher F values indicate stronger effects, whereas values close to 1 suggest no greater explanatory power than expected by chance. In MANCOVA, the F statistic is derived from a multivariate test statistic (here, Pillai’s trace) and evaluated using the F distribution, with statistical significance determined by the associated p-value

The orbitonasal region provided the greatest discrimination between SS patients and controls (Fig. 1B *– IV*). MANCOVA indicated significant differences in facial shape between the groups (*p* < 0.01) even when accounting for age (Table 3). Specifically, SS patients exhibited a tendency toward proptosis, a closed nasofrontal angle, and a shorter nose (Fig. 1B *– V).* Two-block Partial Least Squares (2b-PLS) regression demonstrated a strong covariation between facial shape and age, with correlation coefficients of 0.79 for controls (*p* = 0.001) and 0.85 for SS patients (*p* = 0.001). Theoretical facial shapes were generated for both populations at ages 5, 7, 9, 11, 13, and 16 years, illustrating the relationship between age and shape variation (Fig. 1C). Midfacial hypoplasia, most pronounced from the nasal root to the philtrum, was evident in early childhood but appeared to attenuate by late adolescence. Conversely, ocular protrusion in SS patients was more pronounced than in controls and seemed to worsen with age, in contrast to the improvement seen in midface hypoplasia (Fig. 1C).

Based on linear measurements, SS patients exhibited significant proptosis (*p* < 0.01) and a smaller nasal root height (*p* < 0.001) compared to controls (Table 4).

**Table 4:** Linear measurements obtained from 3D photographs in patients and controls

	Stickler	Controls	*p* value
Patients (*n*)	11	14	
Nasal root height *(mm) Mean ± SD*	36.06 ± 5.0	43.05 ± 6.0	*< 0.01*
Ocular protrusion *(mm)* *Mean ± SD*	17.26 ± 1.9	14.22 ± 1.0	*< 0.001*

**Fig. 5:**
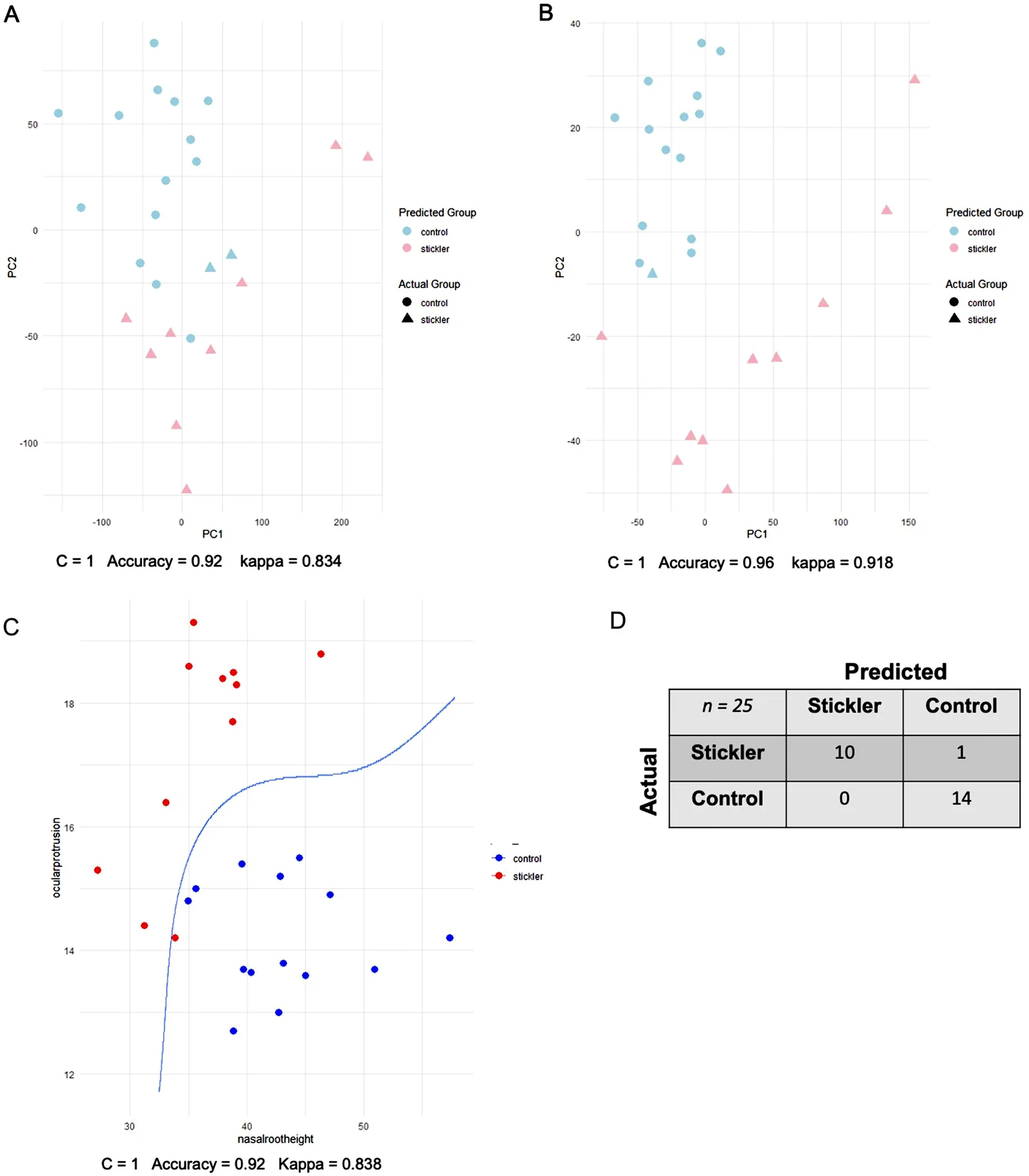
SVM classification performance for Stickler syndrome detection using 3D facial surfaces. (**A**) SVM classification using full facial 3D surfaces. (**B**) SVM classification using orbitonasal 3D surfaces. (**C**) SVM classification plot based on two linear measurements (proptosis and nasal root height). (**D**) Corresponding confusion matrix

Support Vector Machine (SVM) classification models achieved an accuracy of 0.92 when distinguishing SS patients from controls based on full facial surfaces. Two SS patients were misclassified as controls; they carried *COL11A1* and *COL2A1* variants. When the model focused on the orbitonasal region, classification accuracy improved to 0.96, with only one SS patient misclassified (*COL11A1 variant*). The Kappa coefficient was 0.834 for full facial classification and 0.918 for orbitonasal classification (Fig. 5A, B). The parameter C controls the trade-off between maximizing the margin and minimizing the training error. A value of C equal to 1 in a SVM model corresponds to a moderate penalty for misclassification on the training data. It represents a standard compromise. For linear measurements, with C = 1, the model achieved 92% accuracy, and the Kappa coefficient was 0.838 (Fig. 5C, D).

## Discussion

### Early diagnosis in Stickler syndrome

In Europe, hereditary retinal damage is the leading cause of visual impairment and blindness in children [20]. Stickler syndrome is the most common genetic cause of inherited and childhood retinal detachment [3, 21]. The incidence of rhegmatogenous retinal detachment (*caused by vitreous traction*) in SS is estimated at approximately 50% [22–24]. While it most commonly occurs between ages 10 and 30 [5], it is also frequently observed before the age of 10 [6].

Although surgical techniques for retinal detachment repair exist, the failure rate is significantly higher in SS patients than in the general population, with primary success achieved in only 50% of cases [23]. These poor outcomes are largely attributed to the high prevalence of giant retinal tears, delayed detection, and rapid disease progression, resulting in over 20% of SS patients presenting with inoperable retinal detachment [23]. As a result, retinal detachment is the most feared complication of Stickler syndrome, as it is the leading cause of irreversible visual impairment.

When combined with the risk of auditory complications, SS can lead to severe visual, auditory, and speech impairments in young children, resulting in significant lifelong consequences. For patients with an identified SS diagnosis, proactive prevention and close monitoring can effectively reduce the risk of complications and associated morbidity [2, 24, 25]. However, as with many rare diseases presenting with non-specific clinical signs, delayed diagnosis remains a major challenge, particularly in the absence of an associated Pierre Robin sequence [2, 26]. In many cases, SS remains undiagnosed until an ophthalmologic complication arises.

Early screening is therefore a critical challenge in SS. Our research was motivated by the need to establish specific, standardized criteria to assist in the early screening of SS, with the goal of improving prognosis through prophylactic treatments and early follow-up.

### Craniofacial phenotype in stickler syndrome

Distinctive facial features associated with SS have been previously described in the literature [2, 4]. However, these phenotypic traits remain imprecise and are largely based on subjective clinical impressions, making their identification challenging and largely restricted to experienced physicians in rare disease centers.

Controlled Principal Component Analysis (PCA) performed on 3D facial surfaces suggested the presence of a characteristic “Stickler face,” primarily defined by distinctive features in the orbitonasal region: midface retrusion, a depressed frontonasal angle, a less projected nasal tip, and a shorter nose. The observed tendency toward proptosis may be secondary to congenital myopia, a common feature of SS.

Using a controlled AI-based approach on 2D photographs, we identified hypertelorism, thinner and wider palpebral fissures, frontonasal hypoplasia and a longer philtrum as additional distinguishing features. Furthermore the 2D analyses identified a more pronounced facial phenotype in patients harboring a *COL2A1* variant, allowing for clearer discrimination from controls compared with patients carrying a *COL11A1* variant. Both 2D and 3D analyses consistently indicated mild mandibular retrognathia in SS patients, which aligns with its known association with Pierre Robin sequence. According to the 2D analyses, this feature appeared to be more pronounced in patients harboring a *COL11A1* variant.

### Craniofacial growth in stickler syndrome

PLS-2b analysis, incorporating the superposition of theoretical facial shapes for SS and control patients aged 5 to 16 years, revealed a decrease in maxillary retrusion and a progressive worsening of ocular proptosis (Fig. 1C). As previously mentioned, proptosis may be attributed to the progressive elongation of the eyeballs due to the worsening of myopia, a highly prevalent feature in SS.

Similarly, the mild retrognathia observed in younger SS patients appears to lessen over time, with the mandibular position becoming comparable between SS and control adolescents (Fig. 1C). This normalization of mandibular position over time aligns SS with Pierre Robin sequence—though in the latter, catch-up growth remains only partial [27, 28].

While the attenuation of facial anomalies in SS with age has been previously suggested [4], it has never been objectively quantified until now. In clinical practice, these findings further support the need for early screening strategies, as craniofacial distinctive features may become less pronounced over time, potentially complicating late diagnosis.

### New screening tools for stickler syndrome

Three-dimensional (3D) photography is already used in clinical practice [29], particularly in craniofacial surgery, where it is most employed for pre- and postoperative assessments. While its diagnostic applications are still emerging, integrating artificial intelligence (AI) and machine learning techniques holds significant promise in this field [29, 30]. Its rapid acquisition, along with its non-invasive and non-irradiating nature, makes it particularly well-suited for young children.

To evaluate the efficacy of facial morphology-based screening, we employed supervised machine learning classifiers: XGBoost for 2D photographs and Support Vector Machine (SVM) for 3D photographs.

Both models demonstrated high classification accuracy in distinguishing SS patients from controls. Unlike the 2D model, which relied on a large database of photographs, the SVM model achieved high accuracy with a small sample size (25 subjects) for 3D photography. Notably, focusing the SVM model on the orbitonasal region further improved classification accuracy, highlighting the informative power of 3D surfaces over 2D photographs and the clinical significance of the orbitonasal area for SS detection. In contrast, retrognathia does not seem to constitute a decisive discriminative criterion. Also, the apparent increase in corneal projection observed in patients with Stickler syndrome, likely secondary to myopia and and potentially reinforced by global maxillary hypoplasia, should not be considered alone as a discriminative feature, since it can also be observed in non-syndromic myopic children [31]. Rather, its association with the palpebral and nasal regions (including the frontonasal area and the philtrum) defines a region of particular interest for screening based on facial phenotype. Clinical suspicion is further strengthened by the presence of compatible systemic or ophthalmological manifestations and/or a relevant family history. These methods could be valuable for targeted screening, particularly in preschool children with severe congenital myopia, a key risk factor for SS. However, 3D imaging technology is not universally available in pediatric centers in France and abroad, primarily due to its high cost and the need for a dedicated room for static multi-camera systems [29].

To address this limitation, we sought to determine whether simple, accurate clinical measurements within the identified regions of interest could serve as reliable differentiators between SS patients and controls. Establishing accessible clinical criteria would enable all practitioners, including those in centers without 3D imaging, to identify potential SS cases. For this purpose, we applied the SVM model using two clinical measurements: nasal root depth and ocular protrusion, yielding satisfactory results (accuracy = 92%), yet slightly less informative than 3D shape of the orbitonasal region.

One of the limitations of our study concerns the age of the patients. The mean age was 6.9 years for the 2D cohort and 10.5 years for the 3D cohort, which represents a relatively advanced age in the context of early screening. Therefore, these preliminary results will need to be confirmed by further studies conducted in patients ideally of preschool age, in larger cohorts.

### Prenatal diagnosis in stickler syndrome

The application of the algorithm to fetal photographs yielded encouraging preliminary results. These findings suggest that, even at very early developmental stages, the face may already exhibit phenotypic characteristics that could be discriminative for the identification of syndromes with specific craniofacial phenotypes. However, these results must be interpreted with caution. Prenatal diagnosis predominantly relies on *in utero* imaging reconstructions, mainly obtained through ultrasound [32]. In current clinical practice, the interpretation of these reconstructions is performed by experienced practitioners but remains partially subjective. While artificial intelligence, through supervised learning algorithms trained on both healthy and syndromic cohorts, represents a promising avenue for assisting in the prenatal screening of craniofacial malformative syndromes, its clinical applicability remains to be demonstrated. Further studies are therefore required to assess the discriminative performance and generalizability of such algorithms when applied to reconstructed 3D surfaces, which are likely to be phenotypically less precise than high-quality 2D photographs.

The face provides valuable phenotypic information that can aid in the identification of complex malformation syndromes in clinically relevant contexts. This study aimed to characterize the craniofacial features of patients with Stickler syndrome and to evaluate the feasibility of using these features for screening purposes, with the goal of reducing frequent diagnostic delays and preventing early-onset ocular complications and vision loss. Moreover, this pilot study highlights the potential for broader application of AI-based screening approaches for rare diseases with distinctive craniofacial phenotypes.

## Data Availability

The code and data (de-identified participants) generated and/or analyzed during this study are available from the corresponding author upon request.
